# Fingerprint restoration using cubic Bezier curve

**DOI:** 10.1186/s12859-020-03857-z

**Published:** 2020-12-28

**Authors:** Yanglin Tu, Zengwei Yao, Jiao Xu, Yilin Liu, Zhe Zhang

**Affiliations:** 1 Zhuhai People’s Hospital (Zhuhai Hospital Affiliated with Jinan University), No. 79, Kangning Road,Xiangzhou District, Zhuhai , 519000 Guangdong China; 2 Harbin Institute of Technology Shenzhen, HIT Campus of University Town of Shenzhen, Shenzhen, 518055 Guangdong China; 3 Department of Biostatistics, Yale School of Public Health, 60 College Street, P.O. Box 208034, New Haven, CT 06520 USA

**Keywords:** Incomplete fingerprint, Fingerprint description, Reconstruction, Breakpoints, Bezier curve

## Abstract

**Background:**

Fingerprint biometrics play an essential role in authentication. It remains a challenge to match fingerprints with the minutiae or ridges missing. Many fingerprints failed to match their targets due to the incompleteness.

**Result:**

In this work, we modeled the fingerprints with Bezier curves and proposed a novel algorithm to detect and restore fragmented ridges in incomplete fingerprints. In the proposed model, the Bezier curves’ control points represent the fingerprint fragments, reducing the data size by 89% compared to image representations. The representation is lossless as the restoration from the control points fully recovering the image. Our algorithm can effectively restore incomplete fingerprints. In the SFinGe synthetic dataset, the fingerprint image matching score increased by an average of 39.54%, the ERR (equal error rate) is 4.59%, and the FMR1000 (false match rate) is 2.83%, these are lower than 6.56% (ERR) and 5.93% (FMR1000) before restoration. In FVC2004 DB1 real fingerprint dataset, the average matching score increased by 13.22%. The ERR reduced from 8.46% before restoration to 7.23%, and the FMR1000 reduced from 20.58 to 18.01%. Moreover, We assessed the proposed algorithm against FDP-M-net and U-finger in SFinGe synthetic dataset, where FDP-M-net and U-finger are both convolutional neural network models. The results show that the average match score improvement ratio of FDP-M-net is 1.39%, U-finger is 14.62%, both of which are lower than 39.54%, yielded by our algorithm.

**Conclusions:**

Experimental results show that the proposed algorithm can successfully repair and reconstruct ridges in single or multiple damaged regions of incomplete fingerprint images, and hence improve the accuracy of fingerprint matching.

## Background

Biometric recognition technologies have attracted intense attention, and their applications have become widespread [1, 2]. Among all biometric features, fingerprints are highly reliable, and forensic experts adopted them routinely in criminal investigations [3]. The fingerprint verification gains its popularity from universality, uniqueness, persistence, high accuracy, and cost-effectiveness [4, 5]. Scientists achieved substantial progress in fingerprint recognition, and complete fingerprints identification achieves high recognition accuracy. However, many issues are there to be addressed for the identification with damaged fingerprints [6]. The loss of informative features with the damaged fingerprints lead to low recognition accuracy.

Low-quality fingerprints are one of the most important and widely used evidence in judicial and forensic institutions worldwide. It has reported that the percentage of incomplete fingerprint images in the fingerprints database is about 10% [7]. Some information will be erroneously removed as the background of the fingerprint when segmenting a low-quality fingerprint because it cannot be processed efficiently. Simultaneously, problems like breakage, wetting, and corrosion in image information can affect fingerprint segmentation. How to accurately and effectively identify low-quality fingerprints is an urgent problem in the field of fingerprint identification. Therefore, it is necessary to tackle the problems of incomplete fingerprints. At present, the research on low-quality fingerprints mainly focuses on problems such as fingerprint segmentation [8–12] and orientation field reconstruction [13–18]. These algorithms can achieve excellent results for high-quality ordinary fingerprint images. However, the segmentation effect of low-quality fingerprint images is often unsatisfactory, mainly because low-quality fingerprint images usually contain many different types of noise, and the line structure is not apparent.

Incompleteness is one of the most significant features of low-quality fingerprints. There are some methods based on the directional field model, while others are based on deep learning [19–21] to try to repair incomplete fingerprint images. The performance of incomplete fingerprint recognition depends mainly on the effect of fingerprint image enhancement. Therefore, it is essential to effectively repair the damaged regions of fingerprints to improve fingerprint recognition accuracy. At present, researchers mainly restore the damaged region by calculating the direction field. Wang and Hu proposed a fingerprint orientation field model based on the 2D Fourier series expansion and used triangular polynomials to match the fingerprint orientation field. They further combined the topological characteristics of fingerprint ridges to reconstruct the missing direction of local fingerprints [22]. Chen *et al.* proposed constructing a directional field dictionary to reconstruct fingerprint [23]. Zhang *et al.* restored the incomplete region by combining the minutiae with the direction field [24]. These reconstruction works are all based on the orientation field. To our knowledge, there is no research on utilizing curves to restore damaged regions. Perumal V *et al.* proposed using Bezier curves to represent ridges to compress fingerprint images [25]. Inspired by this work, a novel fingerprint restoration method is proposed in this paper. We find the breakpoints of each damaged region and match them according to the relative position of corresponding ridges. Then we utilize the Bezier curve to obtain the restoration curve according to the trend of the relevant ridges. Our experimental results show that the proposed method can successfully restore the damaged regions and improve the accuracy of fingerprint matching.

## Methods

### Data preparation

**Fig. 1:**
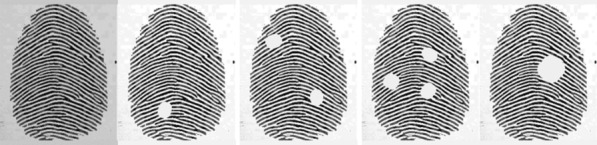
Imnoised fingerprint images.From left to right: original(complete) fingerprint image, fingerprint images one, two, three small-size ecliptic patches, fingerprint image with one large-size ecliptic patch

Five common patterns of fingerprints, namely plain arch, tented arch, left-loop, right-loop, and whorl, were automatically generated by the software SFinGe [26]. We first generated a complete set of 400 fingerprint images containing these five types. To construct a dataset of incomplete fingerprints, we randomly added some elliptic patches of different sizes to the original images so that parts of “damaged” regions were placed on the complete fingerprints, as shown in Fig. 1.

### Fingerprint representation

*Novel fingerprint representation method* In this section, we propose a new fingerprint representation method based on Bezier curves, which can effectively convert the fingerprint image into a series of points, coordinate texts, achieve a better compression effect, and facilitate subsequent follow-up analysis of incomplete fingerprints based on curve fitting algorithms. Fingerprints are made up of numerous ridges, and we fit ridges with Bezier curves. The representation method consists of two steps: fingerprint preprocessing and fitting ridges with cubic Bezier curves.

#### Fingerprint preprocessing

**Fig. 2:**
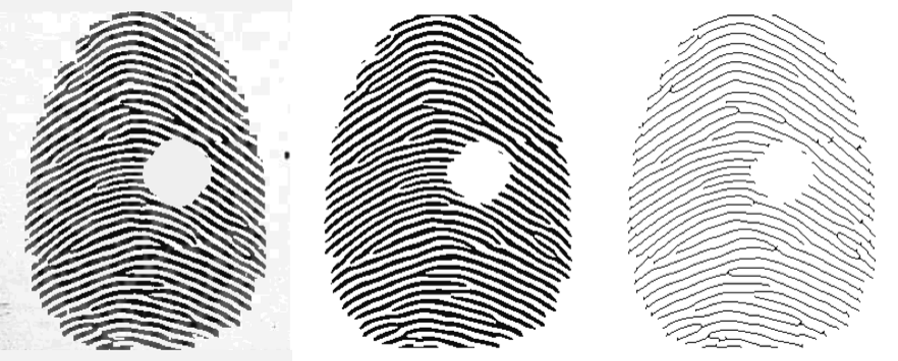
Schematic of preprocessing pipeline.From left to right: original(incomplete) fingerprint image, fingerprint image after gabor enhancement, fingerprint image after thinning

*Fingerprint preprocessing process* The preprocessing converts a fingerprint image into a single-pixel-wide fingerprint skeleton. It contains normalization, Gabor enhancement, binarization, and Thinning, as illustrated in Fig. 2.

- *Normalization* The normalization and equalizing of the fingerprint image are to translate the grayscale mean and variance of the image and convert the grayscale and contrast of the fingerprint image to a pre-specified level. This paper uses dynamic range adjustments for fingerprint equalizing.
- *Gabor enhancement* Gabor enhancement eliminates noise from fingerprint images by the Gabor filter method. Image enhancement way is a manipulation of gray level and the brightness level of the image that it will be useful in image analysis or extraction [27].
- *Binarization* Binarization is to set the pixel value of the grayscale image to binary values to enhance the contrast of the ridge valley.
- *Thinning* The fingerprint image refinement is to process the thick ridgelines’ pixel edges to obtain a fingerprint skeleton with a single-pixel width. It mainly based on the template matching method, which is processed according to the image characteristics of a pixel’s local area (such as 3 × 3, 5 × 5). The template search deletes the line’s edge pixels, making it a single pixel wide.

#### Fitting ridges with cubic Bezier curves

*Introduction to the cubic Bezier curve* A cubic Bezier curve is a parametric curve defined by four points $(x_1,y_1),(x_2,y_2),(x_3,y_3),(x_4,y_4)$. The first and last points are the terminal points of the spline curve, while the other two points are control points that define the slope at the endpoints. Bezier curve fitting process includes :

- *Fingerprint ridge extraction* In this paper, using 8-connectivity neighbors, fingerprint ridges was extracted from the skeleton image, so that all ridges were separated within each fingerprint.
- *Bezier curve fitting* We utilized a cubic Bezier curve to fit every extracted fingerprint ridge. The fitting curve and the original ridge shared the same terminal points. Thus the key in this curve fitting process is to obtain the two control points that define the shape of a Bezier curve. In most cases, to get control points is straightforward. In a complex ridge, we cut it into several segments, and then computed the control points of each segment separately so that a ridge can be expressed with four Bezier control points.
- *Control points* Bezier curves represented all ridges for each fingerprint, and the control points the Bezier curves. Hence, the control points compress the original images.

### Fingerprint restoration

*Reconstruction Algorithm* The control points of the Bezier curve can retain fingerprint ridges. With Bezier curves, we developed a reconstruction algorithm for incomplete fingerprints. The algorithm consists of three parts: damaged region detection, point-match, and restoration Model based on Bezier curves.

#### Detection of damaged regions

**Fig. 3:**
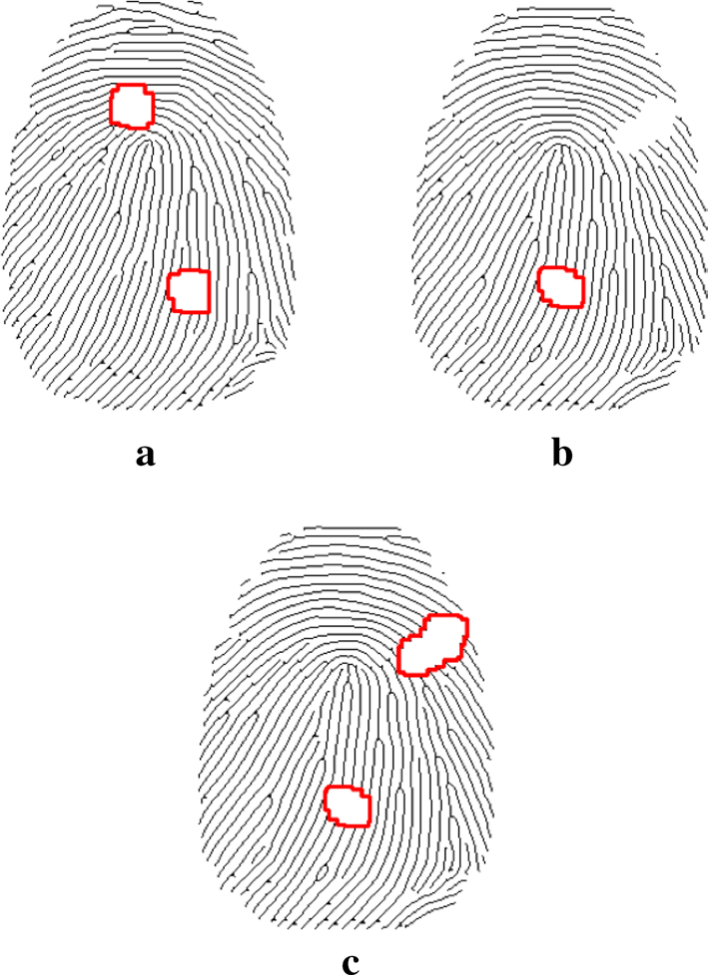
Fingerprint images with detection of damaged regions.a edge detection.b marginal damaged.c detect all damaged regions

*Incomplete area recognition* We employ edge detection to identify incomplete areas, and then group and sort the breakpoints. As shown in Fig. 3a, we can identify the damaged regions (signed in red circle) after applying the edge detection algorithm. However, marginal damaged regions (Fig. 3b) remain undetected. To solve this problem, we applied the edge convex hull detection algorithm on the widened ridges to obtain the external contour, filled the outer outline encircled by external contour with black. Our method can detect all damaged regions - both the inner and the marginal ones(show in Fig.  3c).

#### Breakpoints recognition and mathcing

**Fig. 4:**
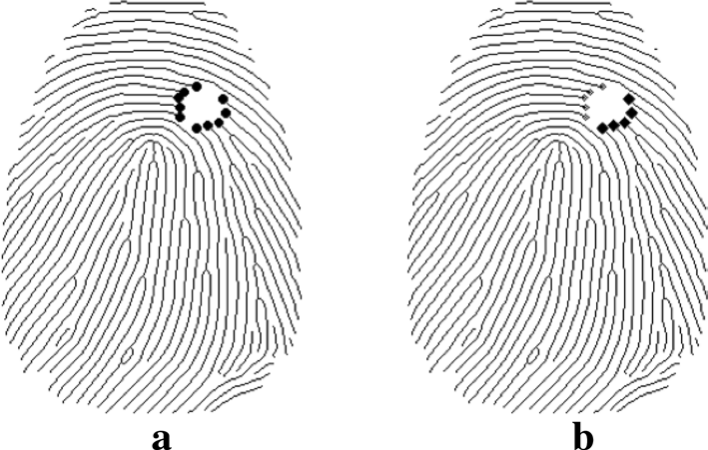
Breakpoints recognition, grouping and sorting.a breakpoints grouping.b breakpoints sorting

*Breakpoint recognition and classification* We identify the end of the ridgeline in the incomplete region as a breakpoint (Fig. 4a). We divided the breakpoints within each damaged region into two groups: the start point and endpoint of the restored segment, as showed in Fig. 4b. Then each group of breakpoints listed in order for the detected edge.

**Fig. 5:**
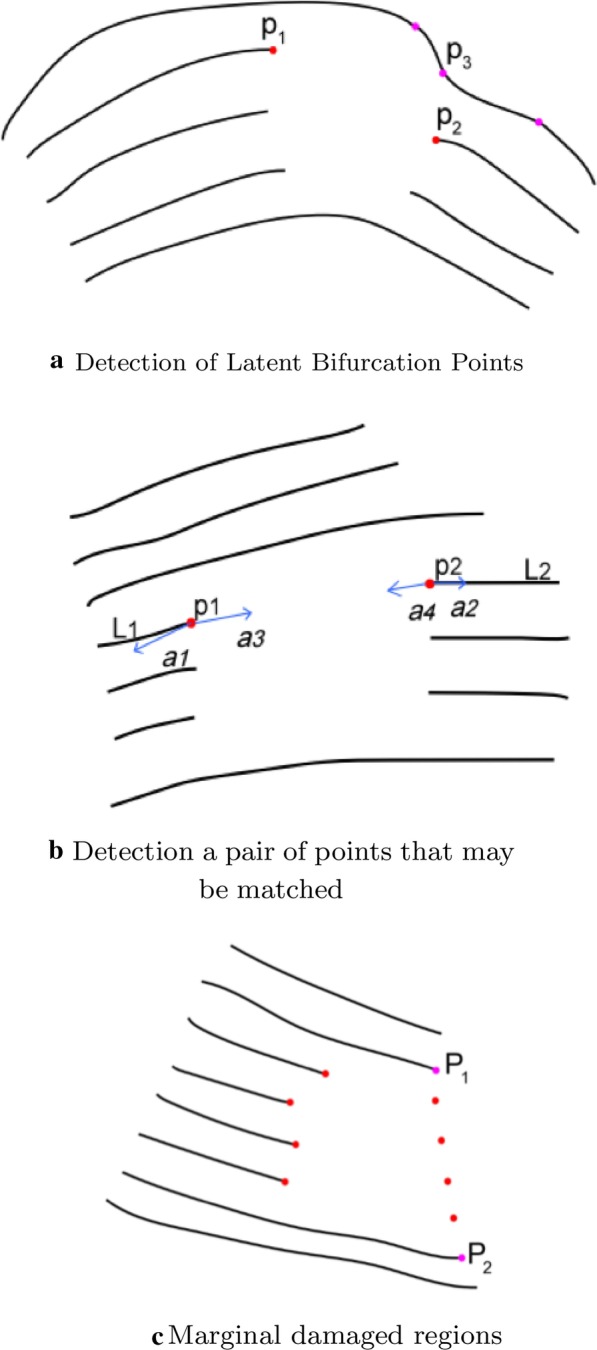
Breakpoints Recognition and mathcing

*Identify potential bifurcation points* Usually, the starting point of each ridge corresponds to an ending point.As shown in Fig. 5a, for edge-breakpoint $p_1$, we search unsmooth points on the edge-ridge (signed in pink) and select the best one as our potential bifurcation point $p_3$ its smoothness and distance from the opposite edge breakpoint $p_2$. However, fingerprint images may contain ridges with bifurcation (branched ridges) [28]. Thus in an incomplete fingerprint image, if a bifurcation point is entirely covered by the damaged region or reserved on the ridge adjacent to the damaged region (partially covered). In this case, one starting point may match multiple ending points.

*Matching Likelihood* We developed a criterion to measure the likelihood of matching two points based on the slopes of corresponding ridges. As shown in Fig. 5b, given two fragmented ridges $L_1,L_2$ with breakpoints $p_1,p_2$, we score the matching $p_1$ with $p_2$.

To define the score function, we use four vectors, $\vec a_1$(from $p_1$), $\vec a_2$(from $p_2$)), $\vec a_3$(from $p_1$ to $p_2$), $\vec a_4$(from $p_2$ to $p_1$), where $\vec a_1$, $\vec a_2$ were used to approximate the tangent line direction of $L_1$, $L_2$ at their breakpoints $p_1,p_2$, and $\vec a_3$, $\vec a_4$ were used to describe the straight line we assumed to connect $p_1, p_2$. We defined a metric (denoted as $\phi$) to measure the likelihood of matching $p_1$ and $p_2$ with angles derived from vectors $\vec a_1$, $\vec a_2$, $\vec a_3$, $\vec a_4$. Smaller value of $\phi$ indicates the higher score of matching $p_1$ with $p_2$. To clarify, we defined $\phi$ as

$$\begin{aligned} \phi (p_1,p_2)=\left\{ \begin{array}{lr} 2\pi -\left\langle \vec a_1,\vec a_3 \right\rangle -\left\langle \vec a_2,\vec a_4 \right\rangle &{} \left\langle \vec a_1,\vec a_2 \right\rangle > \theta \\ \max ({\pi -\left\langle \vec a_1,\vec a_3 \right\rangle , \pi -\left\langle \vec a_2,\vec a_4 \right\rangle }) &{}\left\langle \vec a_1,\vec a_2 \right\rangle \le \theta \end{array} \right. \end{aligned}$$

where $\left\langle \vec a_1,\vec a_2 \right\rangle$ refers to the angle between $\vec a_1, \vec a_2, \left\langle \vec a_1,\vec a_3 \right\rangle$ refers to the angle value between $\vec a_1, \vec a_3, \left\langle \vec a_2,\vec a_4 \right\rangle$ refers to the angle value between $\vec a_2,\vec a_4$. Experimentally, we set $\theta$=165.

*Repair of Marginal damaged regions* In this part, we mainly target the open area located at the fingerprint boundary. In some marginal damaged regions, the marginal ridges were fragmented and are unable to match the breakpoints. As shown in Fig. 5, we need to estimate matching points for the breakpoints based on the number of fragmented ridges. First, we obtained two marginal endpoints $p_1, p_2$ on the edge ridge. We uniformly sampled *n* points (*n* refers to the number of breakpoints) as matching points from the segment $p_1-p_2$. Finally, we matched breakpoints with these bisection points one by one in order.

#### Restoration model based on Bezier curve

*Generation of restoration curve candidates* We assumed that a single cubic Bezier spline could represent the restoration curve. Concretely, we used the breakpoints of matched fragmented ridges as endpoints, estimated control points according to the trend of ridges at endpoints, and generated Bezier spline curves for restoration.

When fixed two endpoints, we move the control point [29] to adjuste the shape of the cubic Bezier curve. Therefore, we generated control points at varying positions and distances concerning the endpoints to obtain a set of curve candidates of different curvature.

**Fig. 6:**
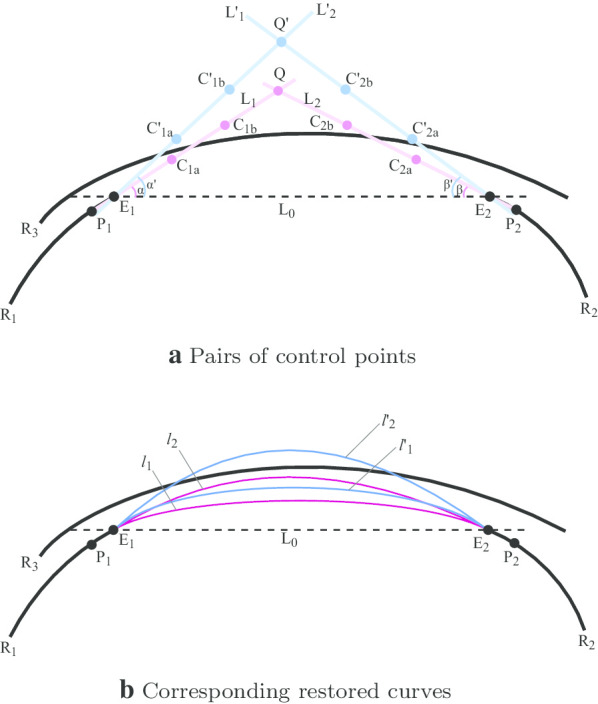
Restoration of Ridges with Bifurcation inside Damagedregions

As shown in Fig. 6a, given two fragmented ridges $R_1, R_2$ whose breakpoints $E_1, E_2$ have been matched in previous steps, we obtained adjacent points of $E_1, E_2$ denoted as $P_1,P_2$, and the adjacent ridge $R_3$, which would later be used as reference to select the best fit among all curve candidates. Then we defined three lines, $L_0, L_1, L_2$ by $E_1$ and $E_2, P_1$ and $E_1, P_2$ and $E_2$, respectively, where $L_1, L_2$ were utilized as our estimations of ending trends of fragmented ridges. The tangent direction at the endpoint of the restoration curve is often determined by the ending trend of the corresponding fragmented ridge. We used $L_1, L_2$ to approximate the tangent lines of $R_1, R_2$ at their breakpoints $E_1, E_2$. Let the intersection and $L_1$ and $L_2$ to be *Q*. Then by selecting points on the line segment defined by $E_1$(or $E_2$) and *Q*, we could generate a set of control points at varying distances from endpoints $(E_1, E_2)$ where each pair of control points would create a restoration curve at distinctive curvature. When using $L_1$ (or $L_2$) as the standard tangent line at the breakpoint for both the fragmented ridge and the restoration curve, we presumed that the original complete ridge is smooth at the breakpoints. In some situations, the original ridge might not be smooth enough at the breakpoints to allow a shared tangent line at breakpoints. To address this situation, we generated new sets of control points in different directions about breakpoints. To describe the relative directions of control points to their respective breakpoints, we computed the angle between $L_0, L_1$ (and $L_0, L_2$). We increased the angle and rotated $L_1, L_2$ for multiple times to new positions, and obtained new sets of control points. With new sets of control points, new restoration curves going into different directions at their start and endpoints can thus be created.

Nevertheless, not all generated restoration curves would be added to the curve candidate set. Thus, for each pair of breakpoints, we could choose one curve as the reference, which is the one with minimal curvature and shared standard tangent lines with the corresponding fragmented ridges Fig. 6b. We would evaluate all the probable curves for its likelihood of being chosen as a suitable restoration.

*Evaluation of restoration curve candidates* Given a set of restoration curve candidates for each matched breakpoints, the mission here is to select the best-fit curve according to the trend demonstrated by the neighboring ridges. To be noted, applying unilateral judgment here may introduce extra bias to the evaluation result. For instance, when the curvature of ridges adjacent to the damaged region shows a significant difference, with either adjacent ridge as a reference, it may turn out that the selected best-fit curves show concordance with the referred ridge, while significant disparity from the other. Thus neighboring ridges at both sides of the given restoration candidate were considered to prevent possible bias caused by unilateral judgment.

**Fig. 7:**
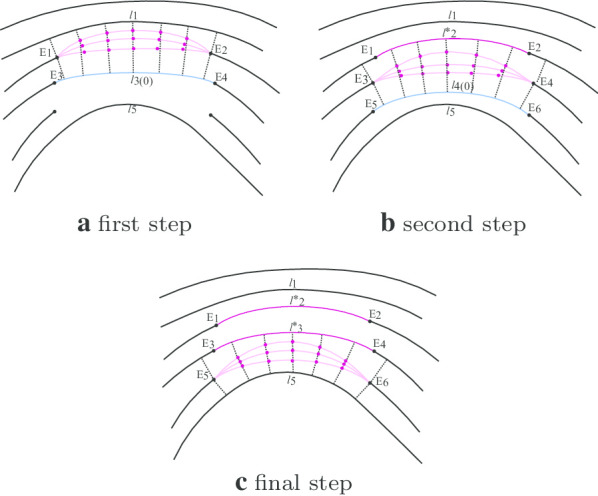
Evaluation process. **a** Repair the first line through evaluation. **b** Repair the second line. **c** Repair the final line

*Evaluation process* The overall evaluation process is as follows. In each damaged region, we conducted this evaluation process from one side to another. To determine the best fit for a given pair of fragmented ridges whose breakpoints were associated with the point-match model, we referred to the pattern of its prior ridge as well as its subsequent ridge. Since the best fit for the next ridge yet to be decided (except that we reached the opposite side of the damaged region), we temporarily used the default case here to evaluate the current set of curve candidates Fig. 7a, the default means that the unrepaired ridge line connected with the set parameters for this evaluation. Once the best-fit curve was chosen for the given fragmented ridge pair, it would then be referred to as the prior ridge for the new evaluation on the subsequent set of curve candidates Fig. 7b. We repeated this process until we reached the other side of the damaged region Fig. 7c. In particular, we defined a distance metric (denoted as $\delta _\ell$) to describe the compatibility of a given curve candidate $\ell$ with its adjacent ridges (or curves). More precisely, for each curve candidate, $\ell$, we uniformly sampled *n* points from the segment and computed their minimal distance to the prior and subsequent ridge, respectively. Then $\delta _\ell$ can be defined as

1 $$\begin{aligned} \delta _\ell =\sum _{i=1}^{n}\Big( | s_i-(s_n-s_1) \cdot \frac{i}{n}-s_1|+|d_i-(d_n-d_1) \cdot \frac{i}{n}-d_1|\Big) \end{aligned}$$

where $s_i$ refers to the minimal distance of the *i*th sampled point to the prior ridge, and $d_i$ refers to the minimal distance of *i*th sampled point to the subsequent ridge. In this study, we set *n* = 5 to calculate $\delta _\ell$. Therefore, our objective was to choose the curve candidate with a minimum $\delta _\ell$ as the best fit for the given pair of fragmented ridges.

*Restoration of ridges with bifurcation* When a branched ridge becomes fragmented, the damaged region can entirely cover the bifurcation point, reserved on the adjacent ridge of the damaged region (latent), or remain completely intact. With bifurcation points detected by applications in “Results” section, we were to generate restoration curves concerning ridges’ intrinsic characteristics with bifurcations. In general, a bifurcation point is also the point of inflection where the ridge becomes unsmooth. Moreover, the branches are convex near the bifurcation point. Therefore, we could no longer apply the same restoration method described in “Evaluation of fingerprin restoration” section. We roughly determined the direction of the restored segment by the ending trend of corresponding fragmented ridges.

**Fig. 8:**
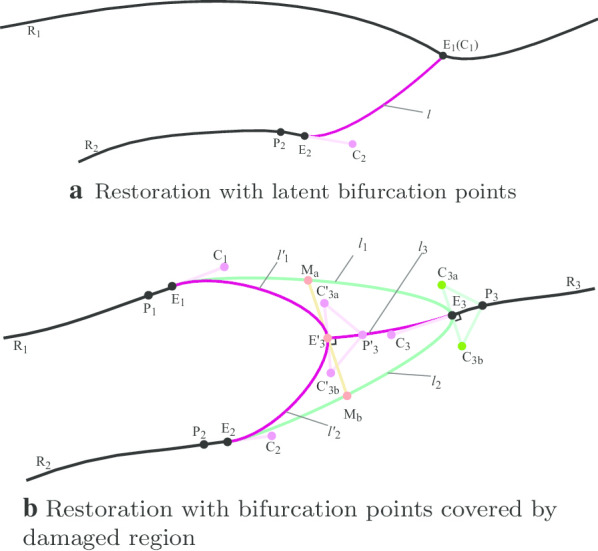
Restoration of Ridges with Bifurcation inside Damagedregions

*Connection of fragmented ridges to latent bifurcation points* When identified a latent bifurcation point by the method claimed in “Results” section, we would then get a ridge $R_1$ adjacent to the damaged region lying the latent bifurcation point, and a fragmented ridge $R_2$ with breakpoint $E_2$ which is recognized as the broken branch at this divergence. The purpose here is to extend $R_2$, reconnect it to the bifurcation point and restore the bifurcate structure. Therefore, we need to get two endpoints and two control points to define a Bezier curve. We considered Ridge $R_1$ as a composition of the main track and one of its branches bonded by the latent bifurcation point. Thus we used the bifurcation point as one endpoint $(E_1)$ of the restoration curve. Moreover, as the trend of the restoration curve at $E_1$ should be distinctive from that of both the main track and the branch, we chose the bifurcation point again as the control point $C_1$. For ridge $R_2$, on the other hand, we found $E_2$’s near neighbor $P_2$ at ridge $R_2$. Then similar to our application in “Evaluation of fingerprin restoration” section, we used the line defined by $P_2,E_2$ as the tangent line for both $R_2$ and the restoration segment, and obtained a control point (denoted as $C_2$) at the line. Consequently, we could generated a restoration curve l defined by endpoints $E_1,E_2$, and control points $C_1,C_2$. Figure 8a.

*Restoration of ridges with bifurcation inside damaged regions* When the damaged region entirely covers a bifurcation point, we cannot get its precise position based on the information of the remaining ridges. Thus we applied a trade-off method here to restore the divergent structure approximately. Accurately, we split the restoration process into two steps: first, we assumed the breakpoint of the main track as the bifurcation point, extended corresponding branches to the point, and obtained two temporary restoration curves $l_1,l_2$. Second, we took the midpoint of the line defined by the midpoints of $l_1,l_2$ as our estimation of the original bifurcation point, and repeated the same restoration process as the first step to generate the final restoration curves. To clarify, as shown in Fig. 8b, we were about to restore a bifurcate structure with three fragmented ridges $R_1,R_2,R_3$, where $R_3$ refers to the main track with $E_3$ as its breakpoint and $R_1,R_2$ refer to the two branches with $E_1,E_2$ as their breakpoints respectively. We got near neighbors $P_1,P_2,P_3$ for $E_1,E_2,E_3$ on their respective ridges, and used auxiliary lines defined by $P_1,E_1$ and $P_2,E_2$ to obtain two control points $C_1,C_2$. Since we would like the two restored curves jointed by the bifurcation point together to make a convex curve, we constructed an auxiliary isosceles right triangle with $P_3$ as its right-angled vertex and $E_3$ as its circumcenter.

We regarded the hypotenuse as the shared tangent line at $E_3$ of the two restoration curves and used the two vertexes of the hypotenuse as two control points. With control points $C_1,C_3a$, endpoints $E_1,E_3$, we could draw curve $l_1$, and with control points $C_2,C_3b$, endpoints $E_2,E_3$, we could draw curve $l_2$. However, based on the assumption that the damaged region entirely covers the original bifurcation point, it is more sensible to search for a point inside the region bounded by $l_1,l_2$ than directly used the breakpoint of the main track as the bifurcation point. Therefore, we performed a trade-off strategy here and obtained the midpoints $M_a,M_b$ of $l_1,l_2$ respectively. Our estimated bifurcation point $E'_3$ lay in the middle of the line segment defined by $M_a,M_b$. Then with the newly estimated bifurcation point, we extended $R_3$ to $E'_3$ and implemented the aforementioned restoration process on $R_1,R_2$, and extended $R_3$.

## Results

### Evaluation of fingerprint description

**Fig. 9:**
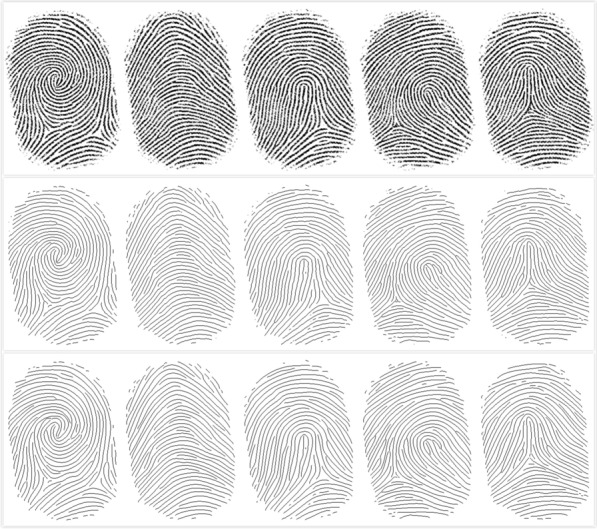
Fingerprint description. The first line, from left to right, is the original fingerprint image of Arch, left-loop, right-loop, tented, and whorl. The second line is the fingerprint skeleton of the preprocessed process corresponding to the first row. The third line, the restored image represented by the Bezier curve

*Compare with the original fingerprint* To verify our proposed representation method’s efficacy, we compared the reconstructed images with the original ones. The fingerprint skeletons of the restored fingerprints highly resemble the corresponding original ones, as shown in Fig. 9. Use our program to compress the complete fingerprint image into a set of Bezier curve control points. Then through our algorithm, reconstruct it into a complete fingerprint and compared the reconstructed images with the original ones. They look very similar visually.

**Fig. 10:**
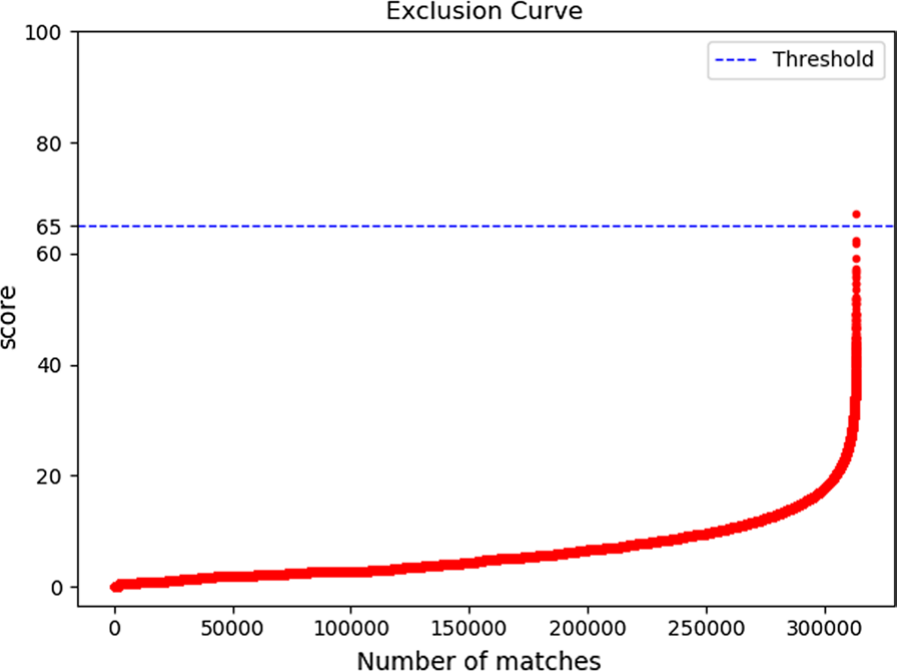
Exclusion curve

**Fig. 11:**
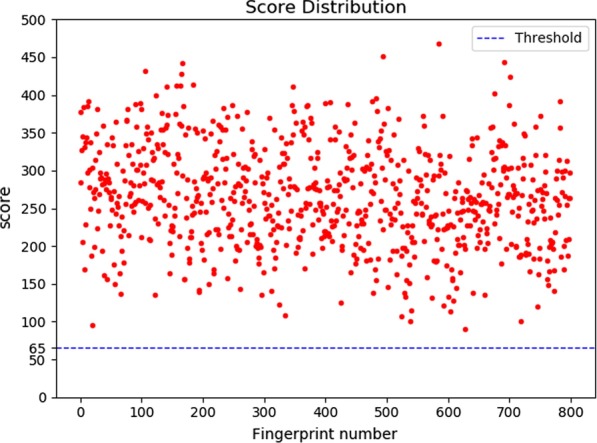
Score distribution

*Match score evaluation* We use fingerprint recognition software recognized in the industry AFISMark to determine whether the fingerprint we reconstructed and the original fingerprint belongs to the same one. Firstly, we matched the existing database to obtain the distribution of scores used to construct an exclusion curve (Fig. 10). We found that most of the matching scores from different fingerprints are below 65, and the false positive rate is $3.16\times 10^{-6}$, so we use 65 as the threshold. Next, we match the reconstructed fingerprint with the original fingerprint and draw the result into a scatter plot (shown in Fig. 11). We can see that all the scores are above the threshold, which indicates that our proposed representation method with cubic Bezier curves can adequately and accurately describe fingerprints.

*Fingerprint compression performance* Besides, we can compress fingerprint images by using representation with Bezier curves. To assess the extent of the original compressed image, we implemented our method on 800 fingerprint images and compared the image sizes before and after the compression. It turns out that the average fingerprint image size before compression is 33.3 KB, while the average fingerprint size after compression is 4.6 KB. More detailed information of five types of fingerprints can be found in Table 1. We can see that the compression has achieved similar performance among fingerprints of various types. However, it is noted that the compression time is relatively long because the fingerprint has undergone a series of preprocessing, which is quite time consuming, while the restoration time is short. In this way, we can describe the Bezier curve fingerprint and effectively compressed the fingerprint image to release the storage space occupied by the fingerprint image.

**Table 1:** Fingerprint compression

Num	Type	Size before compression (KB)	Size after compression (KB)	Compression time (s)	Restore time (s)
1	Arch	33.8	4.1	17.6	1.1
2	Arch	33.5	4.1	15.9	1.1
3	Left-loop	34.5	4.4	18.2	1.0
4	Left-loop	34.3	4.2	18.0	1.2
5	Right-loop	34.2	4.5	17.7	1.1
6	Right-loop	34.1	4.1	17.9	1.2
7	Tented	33.9	4.4	17.6	1.1
8	Tented	33.7	4.7	15.5	1.0
9	whorl	34.5	4.6	19.9	1.0
10	whorl	33.2	4.5	18.0	1.0

### Evaluation of fingerprin restoration

**Fig. 12:**
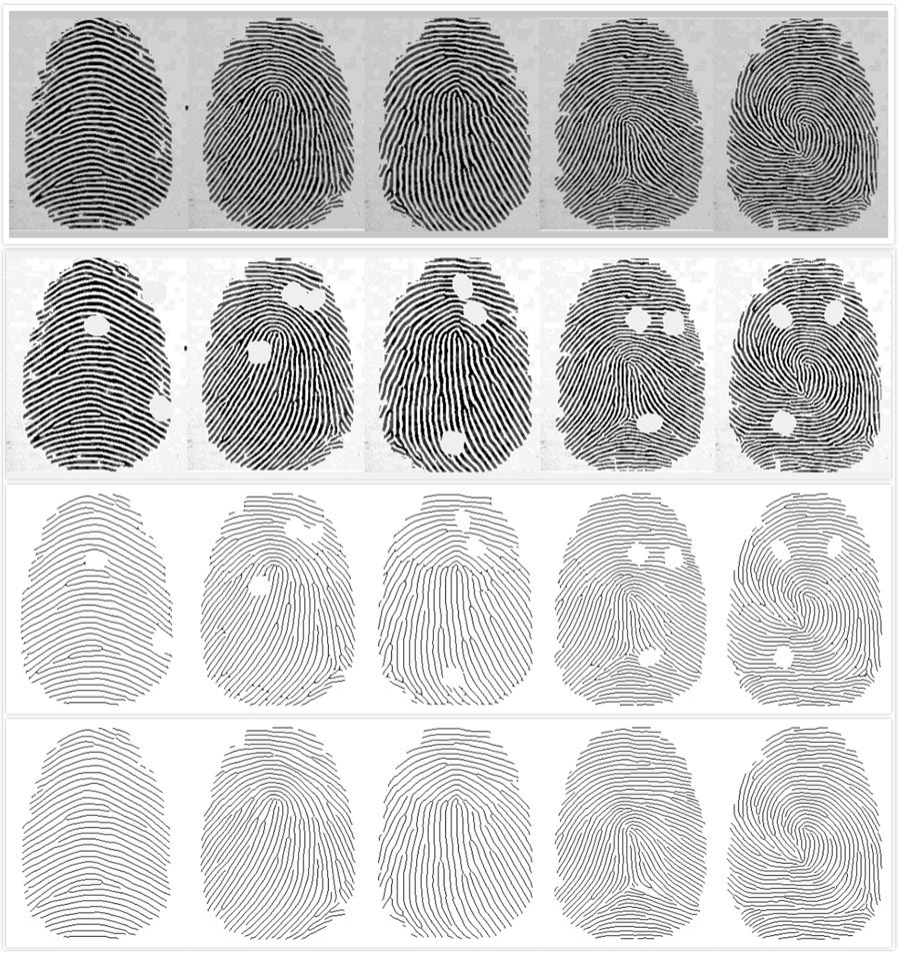
The experimental result for repairing an incomplete image. The first line is five types of original(complete) fingerprint images. The second line is the corresponding incomplete fingerprint image; the third is the fingerprint image with preprocessing; the last is the fingerprint image after restoration

To evaluate our proposed algorithm’s performance, we conducted experiments on the dataset of 400 incomplete fingerprint images (“Background” section) . Figure 12 shows the experimental result for repairing a fingerprint image with two damaged regions using the proposed restoration algorithm. We can see that fragmented ridges restored in multiple damaged regions. Moreover, the trends of restored segments are, in general, concordant with the trends demonstrated by the surrounding ridges.

*Analysis of repair results* To quantitatively evaluate the outcome of the proposed method, we calculated the average matching score of each fingerprint type by the software SourceAFIS, which acquires fingerprint image’s feature construction templates on inputs and generates similarity scores between fingerprint templates on the output [30]. Moreover, we also evaluated the real fingerprint data, which is the FVC2004 DB1 dataset. (Table 2) For a given sample, we computed the similarity score between the imnoised incomplete image and the original complete fingerprint. Thus the ideal score is obtained when we use the original complete fingerprint image to match itself.

**Table 2:** Average matching scores for SfinGe Synthetic dataset and FVC DB1

Types	SfinGe					FVC DB1
	Plain-arch	Left-loop	Right-loop	Whorl	Tented-arch	All
Incomplete	158.37	298.92	247.68	322.57	293.30	242.74
Restored	199.18	339.61	281.62	350.78	315.25	273.24
Original	227.05	401.97	344.85	438.12	428.79	473.3

**Fig. 13:**
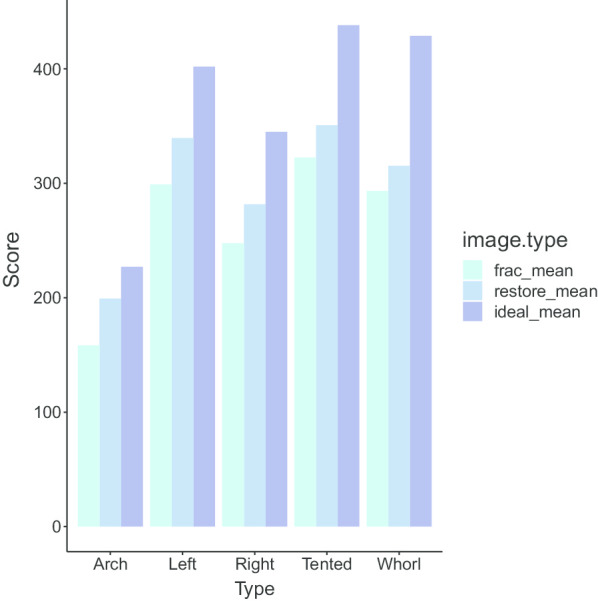
Comparison of scores before and after repair

*Restoration result measurement parameters* As shown in Fig. 13, we can see that for all five types of fingerprint, we improved the average matching score by our restoration. However, the ideal cases in five fingerprint types have different matching scores since scores given by SourceAFIS are affected by the number of minutiae features in fingerprint images. Hence, we defined an improvement ratio *r* as

$$\begin{aligned} r=\frac{S_{restore}-S_{frac}}{S_{ideal}-S_{frac}} \end{aligned}$$

where $S_{restore}$, $S_{frac}$, and $S_{idea}$ refer to the score of restored, incomplete (or fractured), and original fingerprint images of each sample. Then we calculated the average improvement rate ${\bar{r}}$ for each fingerprint type. As shown in Table 3, the proposed restoration method has varied performances among five examined fingerprint types. In general, the ${\bar{r}}$ is between the range 22%-60%. In particular, the proposed method has achieved the highest ${\bar{r}}$ in plain-arch, which is of least complexity among the examined types. By contrast, the restoration method got relatively low values of ${\bar{r}}$ in handling whorl and tented-arch, where much more singular points inside fingerprints greatly increase the complexity. In the FVC DB1 dataset, our algorithm has also improved by 13.22%, indicating that our algorithm performs well on real datasets.

**Table 3:** Average improvement ratio for SfinGe Synthetic dataset and FVC DB1

	SfinGe					FVC DB1
Type	Plain-arch	Left-loop	Right-loop	Whorl	Tented-arch	All
$\bar{r}$	60.57%	46.03%	39.43%	29.33%	22.34%	13.22%

**Fig. 14:**
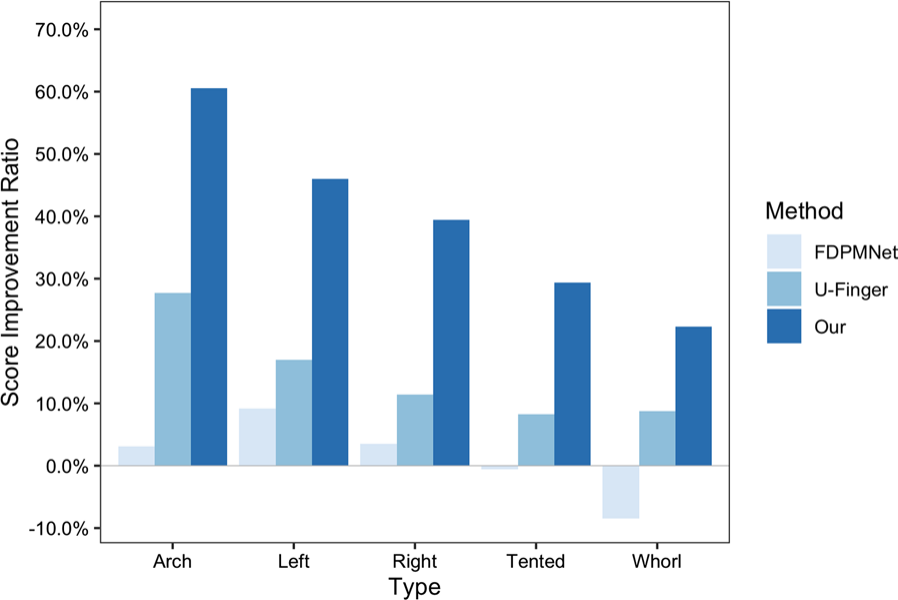
Comparison with U-finger and FDP-M-net

*Compare with others* In order to better illustrate the superiority of our algorithm, we chose several open-source methods for comparison. Since the method based on the traditional direction field is not open source, so we choose the deep learning methods developed rapidly in recent years, U-finger [19] and FPD-M-net [31]. They are both convolutional network models. We tested U-finger and FPD-M-net on the repair match scores of five types of incomplete fingerprints. Figure 14 shows the improvement ratio r of match scores of each type. We can see that the three methods can repair incomplete fingerprint. For arch and left types, the average improvement ${\overline{r}}$ of FDP-M-net is 3.1% and 9.16%, and the ${\overline{r}}$ of U-finger is 27.74% and 16.99%, which are all Significantly lower than our method. In the more complex types of Tented and Whorl, FDP-M-net performs poorly, and the repair result is not as good as the original incomplete fingerprint, which shows that the model cannot handle this type of fingerprint well. Moreover, U-finger can also effectively repair. However, they are less effective than our method. These also show the superiority and robustness of our method in repairing incomplete fingerprints.

**Fig. 15:**
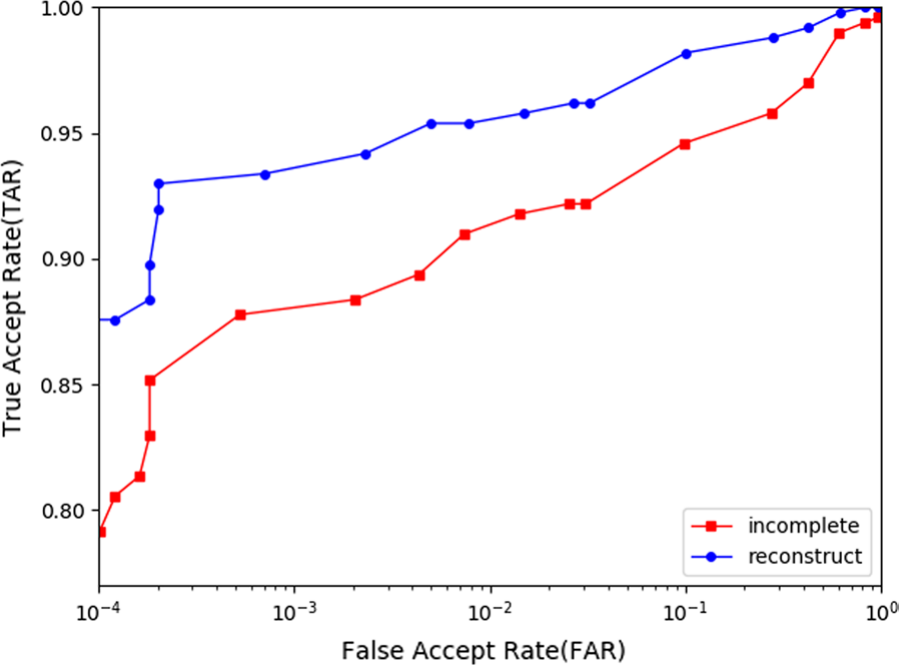
ROC curves with and without proposed method in type-I attack

**Fig. 16:**
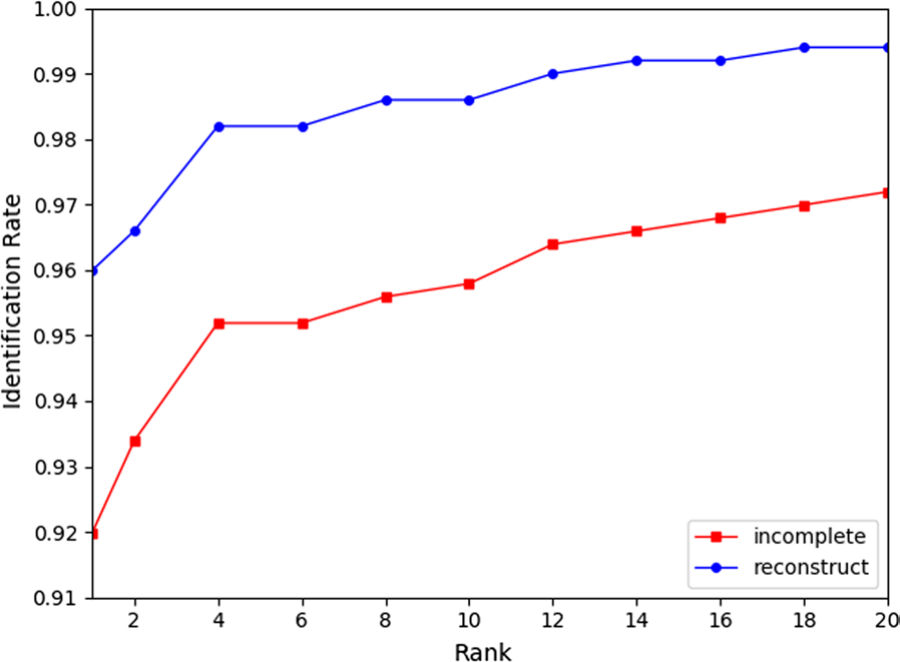
CMC curves with and without proposed method in type-I attack

Besides, we computed the equal error rate(EER), drew Receiver Operating Characteristic (ROC) curves and Cumulative Match Characteristic (CMC) curves in the cases of minutiae-based matching respectively with and without the implementation of our proposed method. As shown in Table 4 , Figs. 15 and 16, an obvious decrease of EER for incomplete fingerprint images from both databases can be observed with the application of our proposed method.

**Table 4:** Matching performance with and without proposed method

Type	SourceAFIS		FVC DB1	
	Without	With	Without	With
$EER (\%)$	6.56	3.79	8.46	7.23
$FMR1000 (\%)$	5.93	1.84	20.58	18.01

## Discussion

Biometric recognition technologies play an essential role in authentication issues. Among all biometric features, fingerprints are routinely adopted in criminal investigations owing to their many desirable characteristics such as universality, persistence, high accuracy, and uniqueness. However, incomplete fingerprints with loss of informative features usually lead to a significant decline in recognition accuracy.

As described in this article, our proposed algorithm can effectively repair incomplete fingerprints and improve the recognition success rate, but there is room for future study and improvement. Our method can be applied to different fingerprint types; the restoration performance varies in dealing with these diverse scenarios. In general, the proposed method performs better in repairing fingerprints with plain and simple patterns (E.g., plain-arch), while the outcome may be less desirable when the patterns become steep and complicated. In the future, we will focus on improving the repair of complex types of fingerprints and a greater degree of incompleteness.

## Conclusion

In this paper, we re-described the fingerprint and proposed a novel algorithm to detect and restore fragmented ridges in fingerprints. We generated a dataset containing 400 images of five common fingerprint types (80 images for each type): plain arch, tented arch, left-loop, right-loop, and whorl. To initialize, we processed all images through fingerprint preprocessing, and thus extracted fingerprint skeleton pixels. Using the obtained fingerprint skeletons as input, we first located damaged regions and built up a “point-match” model, then restored the fragmented ridges based on the Bezier curve. In constructing the “point-match” model, we formulated the likelihood of matching between two detected breakpoints to make a possible connection. To better address different patterns of fragmented ridges, we designed “point-match” and restoration models for branchless ridges, ridges with latent bifurcation points, and ridges with bifurcation points inside damaged regions respectively.

This new fingerprint description method using the Bezier curve can adequately describe the fingerprint image and reduce the size, which is conducive to data storage. We evaluated the performance of the proposed method with matching scores computed by the fingerprint matching software. The experimental results show that the proposed method has achieved higher scores in all concerned fingerprint types in terms of both the average matching score and the average improvement rate (which is a normalized score of improvement), indicating that the proposed algorithm has effectively assisted incomplete fingerprints to achieve better performance in fingerprint matching.

Although there is no quantitative comparison with the traditional direction field method, after analysis, our proposed method has demonstrated the following notable advantages. First, the processing time can be much less since the tedious computation of the orientation field is no longer required. Second, our algorithm can realize the picture to the picture repair process, making repair more convenient. Compared with deep learning-based methods, our method performs well, has good robustness, and has low-performance requirements on the machine.

## Data Availability

The source code and the data are avaliable in the github repository, [https://github.com/tuyanglin/Fingerprint-Restoration].

## Abbreviations

ERR: Equal error rate
FMR: False match rate
FVC: Fingerprint verification competition
DB1: The first database
KB: Kilobyte
ROC: Receiver operating characteristic curves
CMC: Cumulative match characteristic curves
